# The Best Boys’ and Girls’ Magazine

**Published:** 1873-01

**Authors:** 


*The Best Boys' and Girls' Magazine.*

Demorest's Young America is always sparkling with entertaining stories, poems, music, puzzles, games, travels, and other pleasant features, is profusely illustrated, and cannot fail to amuse, instruct and elevate, and assist to make the lives of youthful Americans useful, truthful and happy. The February number, just received, is a real gem. Yearly, one dollar. Address, W. Jennings Demorest, 838 Broadway, New York.

